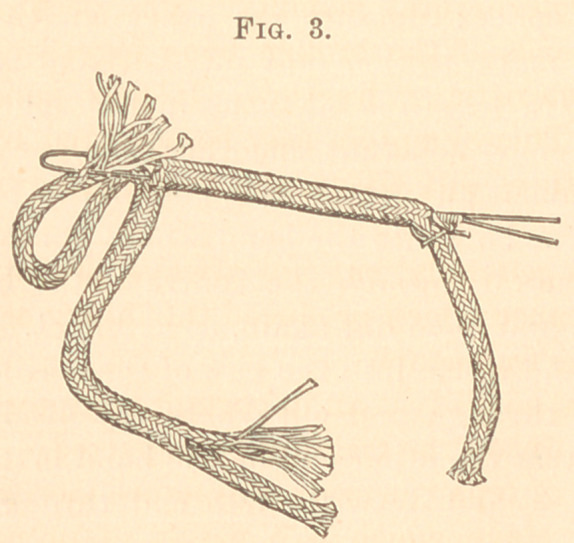# How to Splice Engine Bands

**Published:** 1890-02

**Authors:** George A. Maxfield

**Affiliations:** Holyoke, Mass.


### HOW TO SPLICE ENGINE BANDS.[1]

BY GEORGE A. MAXFIELD, D.D.S., HOLYOKE, MASS.

In thinking over what I could present under the head of Dental Technics, and after extended inquiries among my dental friends, and finding that none of them knew of this method of making a splice, I decided to demonstrate it at this meeting. This method is not original with me,—though I am probably the first to demonstrate it to the profession,—but was invented, I believe, a few years ago by a foreman in one of our woollen-mills at Holyoke, and is now used in most of the mills where a braided band is used. The manner in which most of the dentists splice their bands is, to say the least, a very clumsy one. It takes considerable time to make it, it is not very strong, and never runs smoothly. The splice which I shall show you is made very quickly, makes a strong, even splice, and runs smoothly; in fact, the harder you pull on the band the stronger it holds. The instrument, which I shall call a needle, used in making the splice is made of piano wire, bent in the form of a hair-pin, the free ends inserted in a wooden handle, and fastened so that they will not pull out, allowing the bow end to extend about two and one-half inches from the handle. The sides of the bow must be bent near enough together to allow it to pass easily through the centre of the band.

To make the splice: Measure the exact length the band must be when spliced, mark it, then cut off the band say seven inches longer. This extra length is taken up in the splice. A splice six inches long is stronger and runs smoother than one only four inches long. Unravel about an inch of each end of the band. Take the needle and pass the bow into the band where you have marked the end to be, then pass it through the centre of the band one-half of the extra length, and then out again, as at Fig. 1. Take the other end of the band and insert into the bow of the needle just enough to hold, and pull it through and out where the needle first entered. (See Fig. 2.) Treat the other end of the band in the same way as the first (see Fig. 3) and draw the free end through. Smooth out

[1] Demonstrated at the Union Dental Meeting at Springfield, Mass., October 23, 1889.

Fig. 1.

the splice, and cut the ends so that they will come inside of the band, and your splice is finished. If you wish to make the splice

Fig. 2.

smoother, roll it between two pieces of wood. If your band has a *core*, it requires more painstaking in making the splice; yet it is

Fig. 3.

easily done. First draw out the core from each end the length the splice is to be, say six inches, and so manipulate it as to have the ends come inside the band, exactly where the core has been cut. If you are not particular about this, you will have a weak spot at each end of the splice. If you are particular in splicing this kind of a band, you will hardly be able to detect the splice after it is finished.

## Figures and Tables

**Fig. 1. f1:**
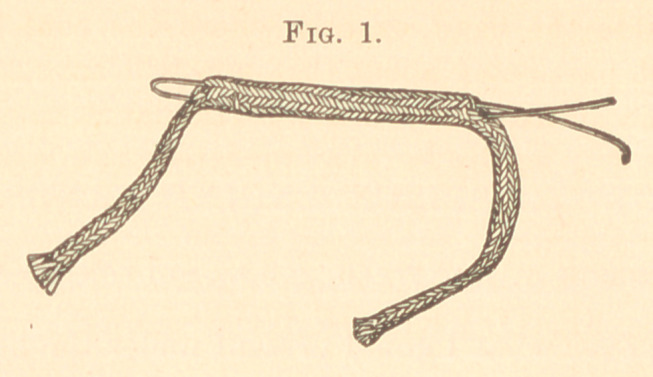


**Fig. 2. f2:**
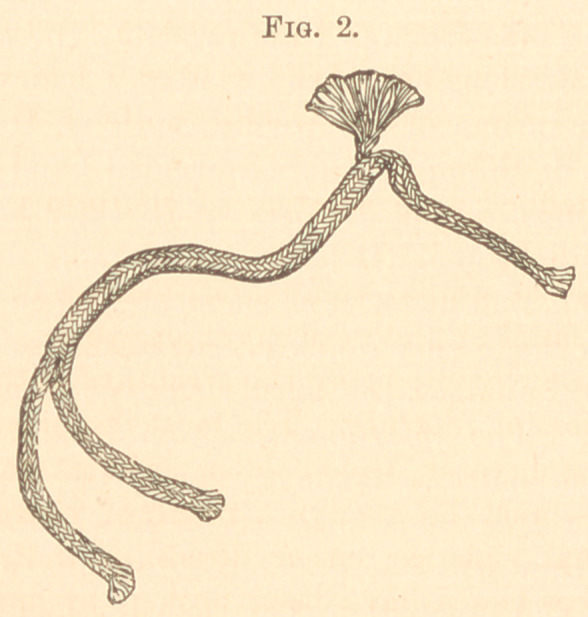


**Fig. 3. f3:**